# Endophytic Fungi from the Cerrado Biome Mitigate Biotic Stress Induced by *Sclerotinia sclerotiorum* in Cotton

**DOI:** 10.3390/plants15081251

**Published:** 2026-04-18

**Authors:** Luciana Cristina Vitorino, Damiana Souza Santos Augusto, Alex Santos Macedo, Marcio Rosa, Fabiano Guimarães Silva, Mateus Neri Oliveira Reis, Marconi Batista Teixeira, Layara Alexandre Bessa

**Affiliations:** 1 Laboratory of Agricultural Microbiology, Instituto Federal Goiano, Rio Verde Campus, Highway Sul Goiana, Km 01, Rio Verde 75901-970, GO, Brazil; alex.macedo@estudante.ifgoiano.edu.br; 2 Laboratory of Metabolism and Genetics of Biodiversity, Instituto Federal Goiano, Rio Verde Campus, Rio Verde 75901-970, GO, Brazil; damianarv2011@gmail.com (D.S.S.A.); fabiano.silva@ifgoiano.edu.br (F.G.S.); mateusnerioliveira@hotmail.com (M.N.O.R.); layara.bessa@ifgoiano.edu.br (L.A.B.); 3 Universidade de Rio Verde (UniRV), Fazenda Fontes do Saber, Rio Verde Campus, Caixa Postal 104, Rio Verde 75901-970, GO, Brazil; marciorosa@unirv.edu.br; 4 Laboratory of Hydraulics and Irrigation, Instituto Federal de Educação, Ciência e Tecnologia Goiano, Rio Verde Campus, Rio Verde 75901-970, GO, Brazil; marconi.teixeira@ifgoiano.edu.br

**Keywords:** *Butia purpurascens*, chlorophyll *a* fluorescence, induced systemic resistance (ISR), microbial antagonism, phytopathology, plant–microbe interactions

## Abstract

The necrotrophic pathogen *Sclerotinia sclerotiorum* compromises the physiological and anatomical integrity of cotton, leading to substantial economic losses due to rapid tissue necrosis, stem blight, boll rot, and leaf wilting. In this context, the use of endophytic microorganisms emerges as a promising strategy for the biocontrol of white mold. This study tested the hypothesis that endophytic fungal strains isolated from the roots of *Butia purpurascens*, a palm tree endemic to the Cerrado biome, could mitigate disease symptoms in *Gossypium hirsutum* L. To evaluate this, cotton plants were subjected to biotic stress imposed by *S. sclerotiorum* to assess the effectiveness of seven fungal strains in attenuating disease. The impact of the pathogen was monitored through growth variables, gas exchange, leaf temperature, chlorophyll *a* fluorescence, antioxidant enzyme activity, proline and malondialdehyde (MDA) levels, and the incidence of rot in petioles, leaves, and flower buds. Overall, inoculation with endophytic fungi significantly alleviated the effects of the phytopathogen, promoting vegetative growth and optimizing physiological performance. Treated plants exhibited alleviated stress in primary photochemistry, reduced non-photochemical energy dissipation, and stable carbon fixation. Additionally, efficient modulation of the antioxidant system and preservation of anatomical structures were observed, minimizing the severe symptoms of white mold. Notably, the non-pathogenic strains BP10EF (*Gibberella moniliformis*), BP16EF (*Penicillium purpurogenum*), and BP33EF (*Hamigera insecticola*) acted as potent physiological modulators, yielding responses similar to those of healthy plants. These results highlight the biotechnological potential of these endophytic strains, which can be explored as both growth promoters and resistance inducers in cotton against white mold.

## 1. Introduction

Among the numerous diseases affecting cotton growth and productivity, Sclerotinia stem rot, caused by the ascomycete *Sclerotinia sclerotiorum* (Lib.) de Bary (Sclerotiniaceae) and widely known as white mold, is one of the most devastating diseases. This pathogen causes billions of dollars in crop losses and is of global economic importance to various agricultural and horticultural crops, including cotton and soybean [1,2]. Infestations are recurrent due to the persistence of *Sclerotinia* species in the soil as mycelia or sclerotia. Dissemination among cotton plants occurs primarily through mechanical transmission, leading to symptoms such as wilting, necrosis, and rotting of stems, bolls, petioles, and leaves [3,4]. Since the fungus infects leaves, flowers, fruits, and stems, this disease poses a severe threat to cotton at all phenological stages [5]. Furthermore, sclerotia are highly resilient multi-hyphal structures capable of surviving for several years in the soil [6]. While ascospores typically colonize senescent flowers to initiate stem infection, infection of other aerial tissues can also occur via wounds or contact with diseased plants [7].

Historically, fungicides have been essential for managing white mold [8]. However, effective control requires applications during critical infestation windows, with the frequency of treatments dictated by the crop’s vegetative period and the availability of petals for ascospore infection [9,10]. Conversely, intensive fungicide use can impair cotton productivity by increasing the production of toxic free radicals [11]. Inappropriate agrochemical application may induce phytotoxicity, manifesting as leaf scorch and reduced photosynthetic capacity, which is vital for boll development [12]. Beyond direct damage, the biological imbalance caused by certain fungicide active ingredients can eliminate beneficial microbiota, triggering secondary pest outbreaks [13,14,15]. Consequently, what is intended as protection can become a technical and financial bottleneck, compromising fiber quality and inflating production costs [16,17]. Moreover, the recurrent use of synthetic fungicides has driven the emergence of resistant *S. sclerotiorum* strains [18,19,20].

An alternative to chemical pesticides is the development of resistant cultivars. However, due to the complex nature of the symptoms caused by this pathogen, genetic breeding programs have faced significant challenges and limited success [21,22,23]. Therefore, developing biological control strategies against this cosmopolitan pathogen is urgent [24]. Aligned with the shift toward sustainable agriculture, the demand for effective biocontrol agents against *S. sclerotiorum* remains a critical gap. Previous studies have shown that the palm *Butia purpurascens*, endemic to the Cerrado biome, harbors multifunctional endophytic fungi [25]. Furthermore, Silva et al. [26] demonstrated that these fungi can alleviate ramulosis symptoms in cotton plants. Thus, we hypothesized that endophytic fungal strains isolated from the roots of this endemic Cerrado plant could alleviate white mold symptoms in cotton.

In recent years, advances in agricultural biotechnology have consolidated the use of biocontrol fungi as cornerstones of integrated pest and disease management [27,28]. Genera such as *Trichoderma*, widely recognized for their efficiency in controlling soil pathogens and promoting root growth [29,30,31,32,33], are joined by entomopathogenic fungi such as *Beauveria bassiana* and *Metarhizium anisopliae*, which offer effective and sustainable alternatives to chemical insect control [34,35]. Silva et al. [36] demonstrated that *Trichoderma* strains can efficiently suppress *S. sclerotiorum* infestations while acting as biostimulants for cotton plants. Despite the growing adoption of these agents, reflecting both scientific evidence of their field efficacy and a response to the need to mitigate resistance in pest populations, the prospecting of novel biocontrol strains against phytopathogenic fungi remains a high demand in the global bioinput market.

In this study, we explored the potential of endophytic strains to mitigate symptoms of wilting and rot in petioles, leaves, and flower buds. Additionally, we evaluated the effects of the interaction between these endophytic fungi and *S. sclerotiorum* on the physiology, antioxidant metabolism, and leaf anatomy of cotton plants. This research sought to identify endophytic fungal strains with antagonistic capacity against *S. sclerotiorum* and to elucidate plant responses during the tripartite interaction between host, endophyte, and phytopathogen. The biocontrol of white mold via endophytic fungi represents an innovative and ecological approach, offering the potential to enhance the sustainability of cotton production and positioning biological control as a strategic element for the economic and environmental viability of modern agriculture.

## 2. Results

**Figure 1:**
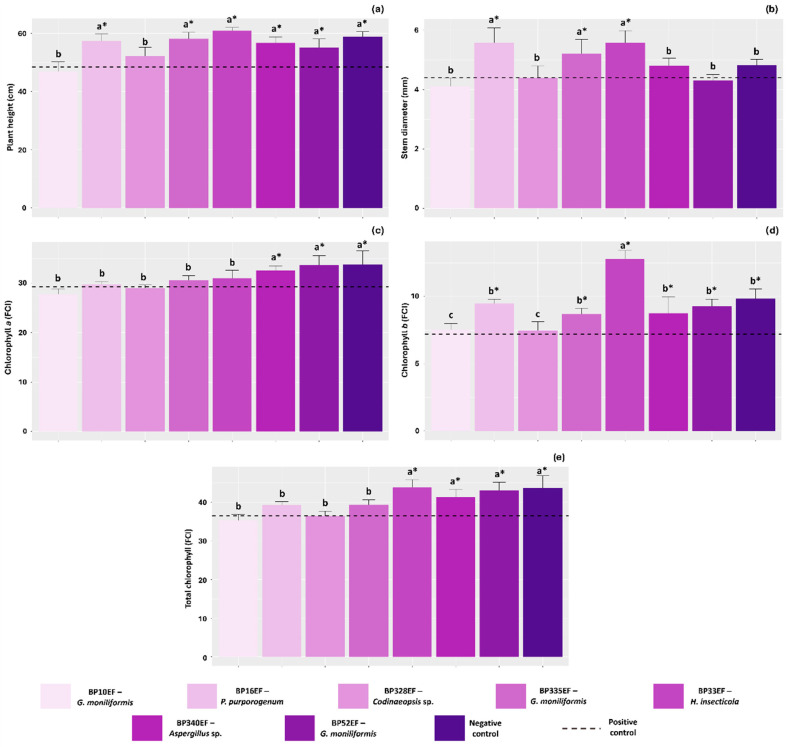
Growth and chlorophyll pigment synthesis in cotton plants (*Gossypium hirsutum* L.) inoculated with endophytic fungi and challenged with *Sclerotinia sclerotiorum*. Plant height (**a**), stem diameter (**b**), chlorophyll *a* index (**c**), chlorophyll *b* index (**d**), and total chlorophyll index (**e**). Negative control corresponds to healthy, non-inoculated plants, and positive control to plants exposed to the phytopathogen without endophytic inoculation. Above the bars, means followed by the same letter do not differ from each other by the Scott–Knott test (*p* < 0.05). * = different from the positive control by Dunnett’s test (*p* < 0.05).

Inoculation with endophytic fungi positively influenced the growth of cotton plants colonized by *S. sclerotiorum*. The greatest plant heights were observed in those treated with strains BP16EF (*P. purpurogenum*), BP335EF (*G. moniliformis*), BP33EF (*H. insecticola*), BP340EF (*Aspergillus* sp.), and BP52EF (*G. moniliformis*), with values of 57.48, 58.10, 60.08, 56.70, and 55.10 cm, respectively, which were comparable to the negative control (healthy, non-inoculated plants) at 58.90 cm (Figure 1a). Stem diameter, however, was significantly greater only in plants protected by endophytes BP16EF (*P. purpurogenum*), BP335EF (*G. moniliformis*), and BP33EF (*H. insecticola*) (5.58, 5.20, and 5.58 mm, respectively), all of which surpassed the negative control (4.82 mm) (Figure 1b). The chlorophyll *a* index was highest in the negative control (33.76 FCI) and in plants inoculated with strains BP340EF (*Aspergillus* sp.) and BP52EF (*G. moniliformis*) under *S. sclerotiorum* stress (32.54 and 33.70 FCI, respectively) (Figure 1c). In contrast, chlorophyll *b* indices were higher in plants treated with strain BP33EF (*H. insecticola*) (2.76 FCI) (Figure 1d). Total chlorophyll levels followed the trends observed for chlorophyll *a* and *b*, peaking in the negative control (43.60 FCI), and in plants exposed to *S. sclerotiorum* but protected by strains BP33EF (*H. insecticola*), BP340EF (*Aspergillus* sp.), and BP52EF (*G. moniliformis*) (43.80, 41.30, and 42.96 FCI, respectively) (Figure 1e).

**Figure 2:**
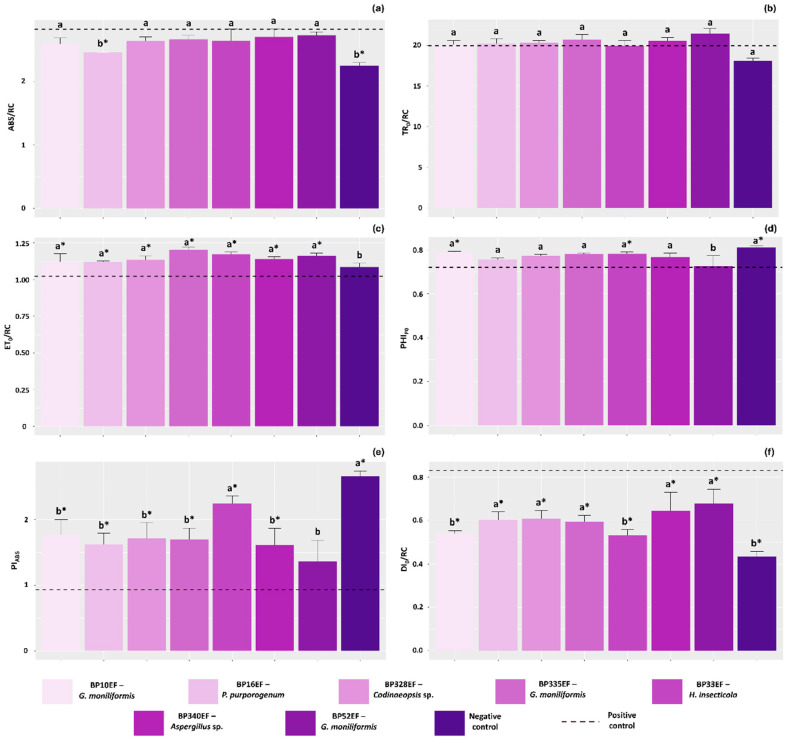
Primary photochemistry parameters in cotton plants (*Gossypium hirsutum* L.) inoculated with endophytic fungi and challenged with *S. sclerotiorum*. Absorption flux per reaction center (ABS/RC) (**a**), trapped energy flux per reaction center at *t* = 0 (TR_0_/RC) (**b**), electron transport flux per reaction center at *t* = 0 (ET_0_/RC) (**c**), maximum quantum yield of primary photochemistry (PHI_P0_) (**d**), photosynthetic performance index (PI_ABS_) (**e**), and specific energy dissipation flux per reaction center (DI_0_/RC) (**f**). Negative control corresponds to healthy, non-inoculated plants, and positive control to plants exposed to the phytopathogen without endophytic inoculation. Above the bars, means followed by the same letter do not differ from each other by the Scott–Knott test (*p* < 0.05). * = different from the positive control by Dunnett’s test (*p* < 0.05).

As expected, the absorption flux per reaction center (ABS/RC) was lowest in the negative control (2.24). Notably, plants inoculated with BP16EF (*P. purpurogenum*) and exposed to *S. sclerotiorum* exhibited values similar to the negative control (2.45), indicating reduced stress levels in the primary photochemistry of these plants (Figure 2a). Regarding the trapped energy flux per reaction center (TR_0_/RC), no significant differences were observed among the inoculation treatments or between them and the positive control (Figure 2b). Overall, the electron transport flux per reaction center (ET_0_/RC) was higher in plants inoculated with endophytic strains compared to the negative and positive controls, which showed lower values (108.50 and 102.34, respectively) (Figure 2c).

The non-inoculated negative control plants, as well as those treated with strains BP10EF (*G. moniliformis*) and BP33EF (*H. insecticola*), showed the highest maximum quantum yield of primary photochemistry (PHI_P0_) (0.80, 0.78, and 0.77, respectively) (Figure 2d). The photosynthetic performance index (PI_ABS_) was lowest in the positive control plants (0.93). Conversely, the negative control and plants subjected to *S. sclerotiorum* but treated with BP33EF (*H. insecticola*) achieved the highest photosynthetic performance (2.65 and 2.24, respectively) (Figure 2e). Consequently, the specific energy dissipation flux (DI_0_/RC) was highest in the positive control (0.83) and significantly lower in the negative control and plants treated with BP10EF (*G. moniliformis*) and BP33EF (*H. insecticola*) (0.43, 0.55, and 0.53, respectively) (Figure 2f).

**Figure 3:**
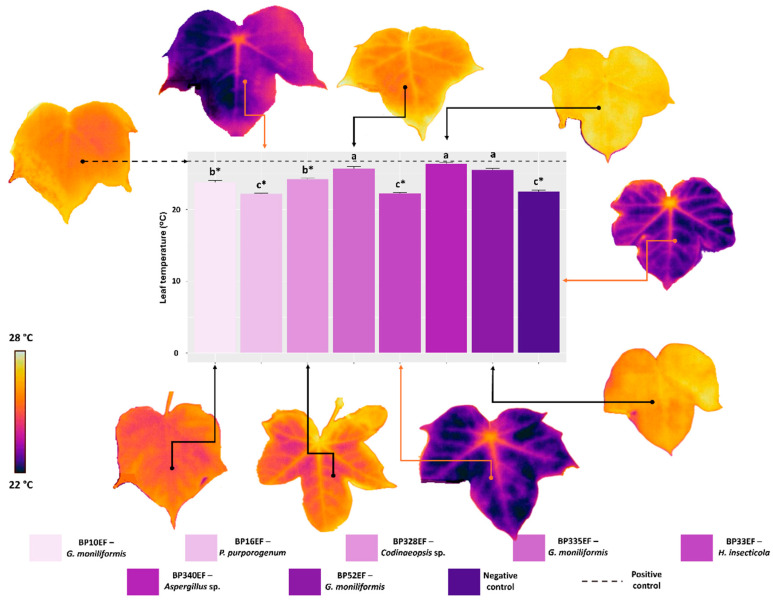
Leaf temperature in cotton plants (*Gossypium hirsutum* L.) inoculated with endophytic fungi and challenged with *S. sclerotiorum*. The images of the leaves were obtained using a thermal camera. Negative control corresponds to healthy, non-inoculated plants, and positive control to plants exposed to the phytopathogen without endophytic inoculation. Above the bars, means followed by the same letter do not differ from each other by the Scott–Knott test (*p* < 0.05). * = different from the positive control by Dunnett’s test (*p* < 0.05).

Leaf temperature measurements revealed a direct relationship with DI_0_/RC. Higher temperatures were recorded in the positive control (26.75 °C) and in stressed plants inoculated with BP335EF (*G. moniliformis*), BP340EF (*Aspergillus* sp.), and BP52EF (*G. moniliformis*) (25.72, 26.40, and 25.22 °C, respectively). In contrast, plants inoculated with BP16EF (*P. purpurogenum*) and BP33EF (*H. insecticola*) exhibited reduced leaf temperatures (22.20 and 22.25 °C), which were comparable to the values observed in the negative control (non-stressed plants, 22.52 °C) (Figure 3).

**Figure 4:**
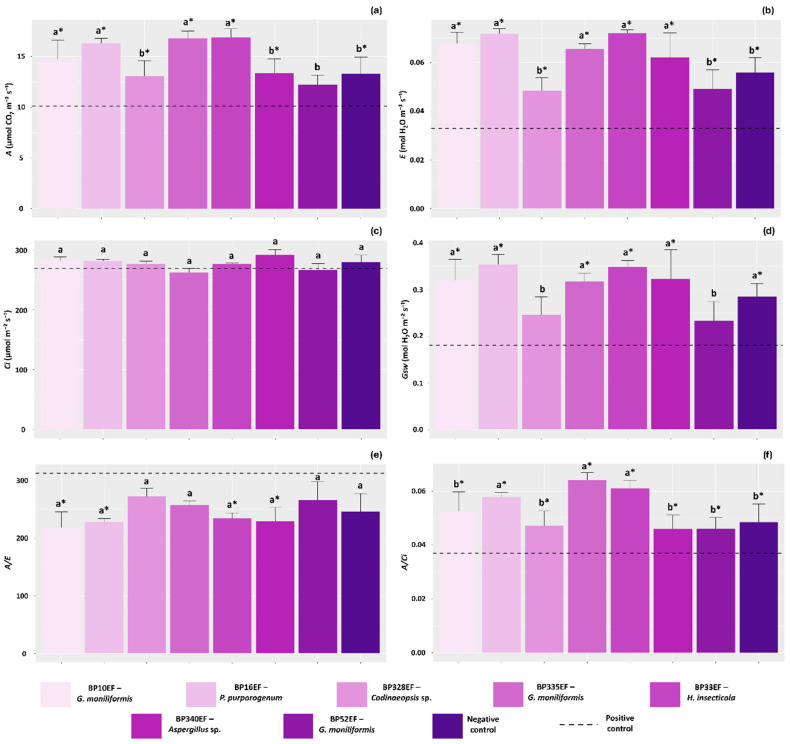
Gas exchange parameters in cotton plants (*Gossypium hirsutum* L.) inoculated with endophytic fungi and challenged with *S. sclerotiorum*. Photosynthetic rate (*A*) (**a**), transpiration rate (*E*) (**b**), internal CO_2_ concentration (*Ci*) (**c**), stomatal conductance (*Gsw*) (**d**), water use efficiency (*A/E*) (**e**) and carboxylation efficiency (*A/Ci*) (**f**). Negative control corresponds to healthy, non-inoculated plants, and positive control to plants exposed to the phytopathogen without endophytic inoculation. Above the bars, means followed by the same letter do not differ from each other by the Scott–Knott test (*p* < 0.05). * = different from the positive control by Dunnett’s test (*p* < 0.05).

Overall, plants treated with endophytic fungi exhibited higher net photosynthetic rates (*A*) than those observed in the positive control (10.12 µmol CO_2_ m^−2^ s^−1^). Among the treatments, the highest average *A* values were recorded in plants inoculated with endophytes BP10EF (*G. moniliformis*), BP16EF (*P. purporogenum*), BP335EF (*G. moniliformis*) and BP33EF (*H. insecticola*) (14.71, 16.32, 16.80, and 16.90 µmol CO_2_ m^−2^ s^−1^, respectively) (Figure 4a). Conversely, transpiration rates (*E*) were significantly lower in stressed, non-protected plants (0.032 mol H_2_O m^−2^ s^−1^). Plants exposed to the pathogen but inoculated with BP10EF (*G. moniliformis*), BP16EF (*P. purporogenum*), BP335EF (*G. moniliformis*), BP33EF (*H. insecticola*) and BP340EF (*Aspergillus* sp.) showed the highest transpiration rates (0.067, 0.071, 0.065, 0.072, and 0.062 mol H_2_O m^−2^ s^−1^, respectively) (Figure 4b). No significant differences were found for internal carbon concentration (*Ci*) among the inoculation treatments or between them and the controls (Figure 4c).

Stomatal conductance (*Gsw*) was also affected by the inoculation treatments, with significant reductions observed in plants stressed by *S. sclerotiorum* (0.18 mol H_2_O m^−2^ s^−1^) and in those inoculated with the endophytes BP328EF (*Codinaeopsis* sp.) and BP52EF (*G. moniliformis*) (0.24 and 0.23 mol H_2_O m^−2^ s^−1^, respectively) (Figure 4d). Due to the lower average *E* values in the positive control plants, the water-use efficiency (*A/E*) was higher in these individuals, as well as in those with high *Gsw* that were inoculated with BP328EF (*Codinaeopsis* sp.) and BP52EF (*G. moniliformis*) (271.41 and 265.20, respectively) (Figure 4e). Furthermore, carboxylation efficiency (*A/Ci*) was significantly lower in the positive control (0.037) and higher in plants protected with strains BP16EF (*P. purpurogenum*), BP335EF (*G. moniliformis*), and BP33EF (*H. insecticola*) (0.057, 0.064, and 0.061, respectively) (Figure 4f).

**Figure 5:**
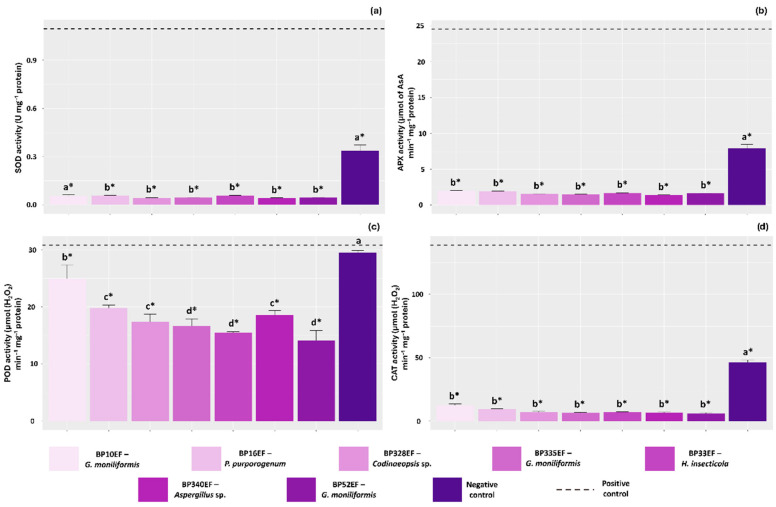
Activity of antioxidant system enzymes in cotton (*Gossypium hirsutum* L.) plant leaves inoculated with endophytic fungi and challenged with *S. sclerotiorum*. Superoxide dismutase (SOD) (**a**), ascorbate peroxidase (APX) (**b**), guaiacol peroxidase (POD) (**c**) and catalase (CAT) (**d**). Negative control corresponds to healthy, non-inoculated plants, and positive control to plants exposed to the phytopathogen without endophytic inoculation. Above the bars, means followed by the same letter do not differ from each other by the Scott–Knott test (*p* < 0.05). * = different from the positive control by Dunnett’s test (*p* < 0.05).

Leaves of plants stressed by *S. sclerotiorum* without endophytic protection exhibited significantly higher activities of the antioxidant enzymes SOD, APX and CAT (0.10 U mg^−1^ protein, 24.52 µmol of Asa min^−1^ mg^−1^ protein, and 138.74 µmol (H_2_O_2_) min^−1^ mg^−1^ protein, respectively) compared to all other treatments. Endophytic inoculation significantly reduced SOD, APX, and CAT activities in cotton leaves; notably, these levels were even lower than those recorded in the negative control (0.033 U mg^−1^ protein, 7.90 µmol of Asa min^−1^ mg^−1^ protein, and 46.25 µmol (H_2_O_2_) min^−1^ mg^−1^ protein, respectively) (Figure 5a, Figure 5b and Figure 5d). POD activity was also highest in the stressed positive control (30.78 µmol (H_2_O_2_) min^−1^ mg^−1^ protein), although it remained similarly high in the negative control (29.51 µmol (H_2_O_2_) min^−1^ mg^−1^ protein) (Figure 5c).

**Figure 6:**
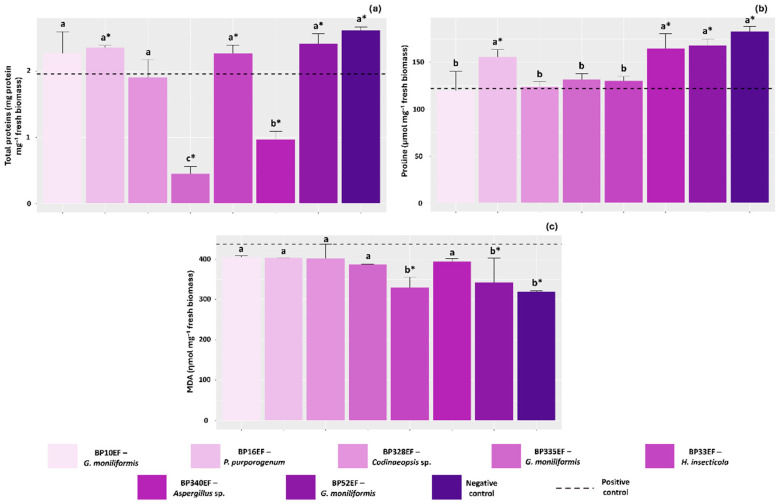
Synthesis of proteins, proline and lipid peroxidation in cotton plants (*Gossypium hirsutum* L.) inoculated with endophytic fungi and challenged with *S. sclerotiorum*. Total proteins (**a**), proline (**b**) and malondialdehyde (MDA) (**c**). Negative control corresponds to healthy, non-inoculated plants, and positive control to plants exposed to the phytopathogen without endophytic inoculation. Above the bars, means followed by the same letter do not differ from each other by the Scott–Knott test (*p* < 0.05). * = different from the positive control by Dunnett’s test (*p* < 0.05).

Total soluble protein content was influenced by endophytic inoculation, with lower concentrations recorded in plants treated with strains BP335EF (*G. moniliformis*) and BP340EF (*Aspergillus* sp.) (0.44 and 0.96 mg protein mg^−1^ fresh biomass, respectively). In the positive and negative controls, higher protein concentrations appeared to be directly associated with the increased synthesis of antioxidant enzymes (1.96 and 2.61 mg protein mg^−1^ fresh biomass, respectively) (Figure 6a). The negative control plants, as well as stressed plants inoculated with BP16EF (*P. purpurogenum*), BP340EF (*Aspergillus* sp.), and BP52EF (*G. moniliformis*), exhibited the highest proline accumulation (183, 155, 164, and 168 µmol mg^−1^ fresh biomass) (Figure 6b). Regarding lipid peroxidation, malondialdehyde (MDA) levels were significantly lower in the negative control (318.37 ηmol mg^−1^ fresh biomass) and in plants inoculated with BP33EF (*H. insecticola*) and BP52EF (*G. moniliformis*) (329.74 and 341.88 ηmol mg^−1^ fresh biomass, respectively). These values were lower than those observed in all other inoculation treatments and the positive control (436.53 ηmol mg^−1^ fresh biomass), indicating reduced damage to cell membranes in these plants (Figure 6c).

**Figure 7:**
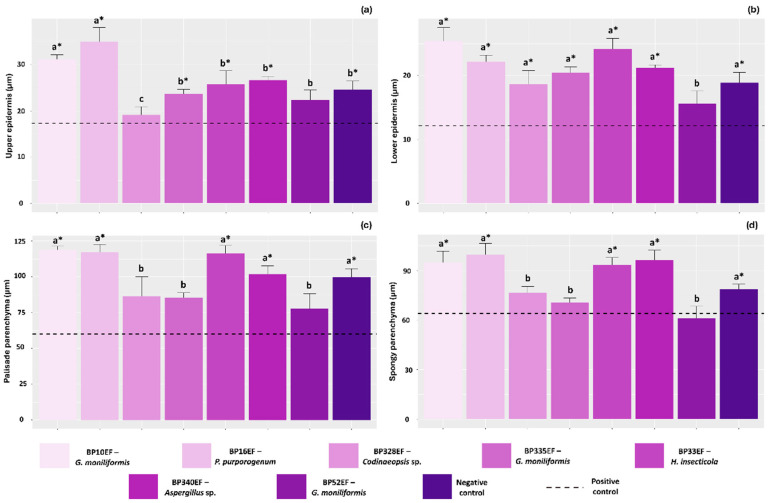
Anatomical development of cotton (*Gossypium hirsutum* L.) plant leaf tissues inoculated with endophytic fungi and challenged with *S. sclerotiorum*. Upper epidermis (**a**), lower epidermis (**b**), palisade parenchyma (**c**) and spongy parenchyma (**d**). Negative control corresponds to healthy, non-inoculated plants, and positive control to plants exposed to the phytopathogen without endophytic inoculation. Above the bars, means followed by the same letter do not differ from each other by the Scott–Knott test (*p* < 0.05). * = different from the positive control by Dunnett’s test (*p* < 0.05).

Overall, plants stressed by *S. sclerotiorum* in the positive control exhibited compromised leaf tissue development, showing lower average thickness for the upper and lower epidermis, as well as for the palisade and spongy parenchyma (17.33, 12.18, 60.15, and 63.99 µm, respectively). In contrast, significantly thicker upper epidermises were observed in plants inoculated with strains BP10EF (*G. moniliformis*) and BP16EF (*P. purpurogenum*) (31.15 and 35.02 µm, respectively). The lower epidermis was better developed in the negative control and in plants subjected to endophytic inoculation, except for the BP52EF (*G. moniliformis*) treatment, which showed averages similar to the negative control (15.62 µm) (Figure 7a,b). The development of the palisade and spongy parenchyma followed a similar trend to that observed for the lower epidermis. The greatest thicknesses for these anatomical structures were recorded in the negative control and in plants inoculated with the endophytic fungi BP10EF (*G. moniliformis*), BP16EF (*P. purpurogenum*), BP33EF (*H. insecticola*), and BP340EF (*Aspergillus* sp.), with values of 118.81, 117.31, 116.42, 101.76, and 99.65 µm for the palisade parenchyma, and 94.88, 99.71, 93.27, 96.40, and 78.51 µm for the spongy parenchyma, respectively (Figure 7c and Figure 7d).

**Figure 8:**
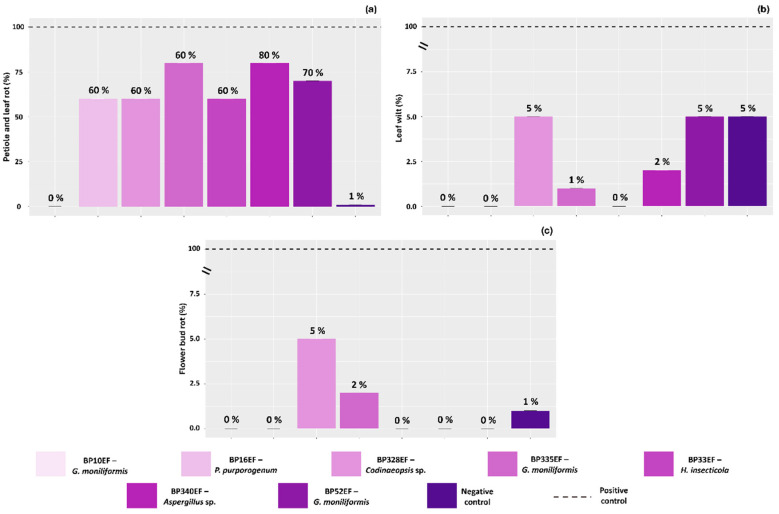
Percentage of symptoms associated with white mold in cotton plants (*Gossypium hirsutum* L.) inoculated with endophytic fungi and challenged with *S. sclerotiorum*. Petiole and leaf rot (**a**), leaf wilt (**b**) and flower bud rot (**c**). Negative control corresponds to healthy, non-inoculated plants, and positive control to plants exposed to the phytopathogen without endophytic inoculation. Percentages were calculated based on the total number of leaves, flower buds, and flowers observed across five replicates (five pots per treatment), with each replicate consisting of two plants per pot.

All plants in the stressed positive control (100%) exhibited characteristic white mold symptoms on petioles, leaves, and flower buds. In contrast, inoculation with endophytic strains significantly reduced the percentage of symptomatic plants. Among the inoculated treatments, the highest control efficiency for petiole and leaf rot was achieved with strain BP10EF (*G. moniliformis*), which resulted in 0% incidence of these symptoms (Figure 8a). Similarly, leaf wilting was effectively suppressed by the endophytic strains, with incidence rates not exceeding 5%. The most effective biocontrol of wilting was observed in plants inoculated with BP10EF (*G. moniliformis*), BP16EF (*P. purpurogenum*), and BP33EF (*H. insecticola*) (Figure 8b). Regarding flower bud rot, while the positive control showed 100% incidence, mortality was drastically reduced in plants treated with BP328EF (*Codinaeopsis* sp.) (5%) and BP335EF (*G. moniliformis*) (2%), compared to 1% in the negative control (Figure 8c).

**Figure 9:**
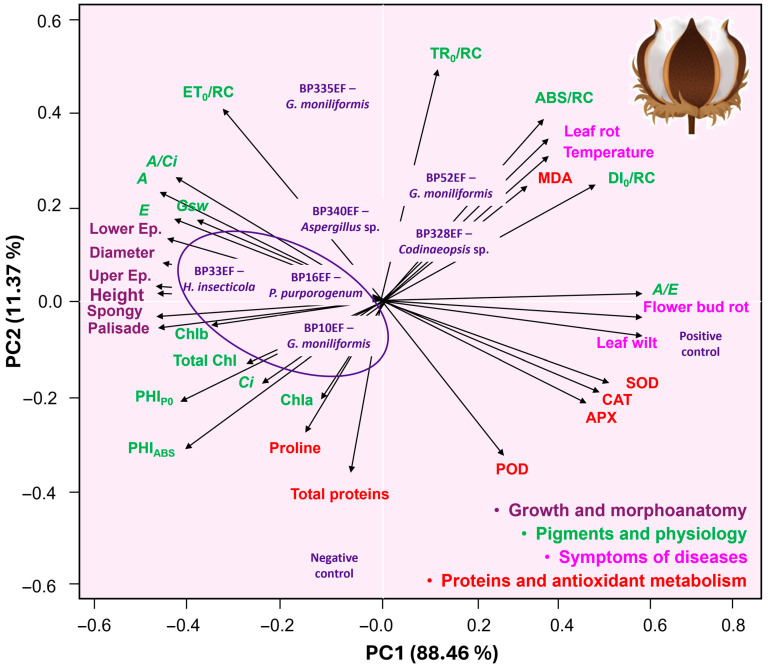
Principal Component Analysis for plant growth data, leaf morphoanatomy, synthesis of photosynthetic pigments, physiology (gas exchanges and fluorescence of chlorophyll *a*), leaf temperature, protein synthesis and oxidative metabolism in cotton plants (*Gossypium hirsutum* L.) inoculated with endophytic fungi and challenged with *S. sclerotiorum*. Epidermis: Ep., chlorophyll *a*: Chla, chlorophyll *b*: Chla, total chlorophyll: total Chl, Net photosynthetic rate: *A*, transpiration rate: *E*, internal concentration of CO_2_: *Ci*, stomatal conductance: *Gsw*, carboxylation efficiency: *A/Ci*, light absorption flux per active reaction center: ABS/RC, electron transport flux per reaction center (ET_0_/RC) at *t* = 0; trapped energy flux per reaction center (TR_0_/RC) at *t* = 0; specific energy dissipation flux (DI_0_/RC), maximum quantum yield of primary photochemistry: PHI_P0_, photosynthetic performance index: PHI_ABS_, catalase: CAT, peroxidase: POD, superoxide dismutase: SOD, ascorbate peroxidase: APX, and malondialdehyde: MDA.

PCA revealed that strains BP10EF (*G. moniliformis*), BP16EF (*P. purpurogenum*), and BP33EF (*H. insecticola*) formed a distinct cluster (Figure 9), which was associated with the highest means for growth and morphoanatomical variables, photosynthetic pigment synthesis, net photosynthesis (*A*), quantum yield (PHI_P0_), and photosynthetic performance (PI_ABS_). Conversely, the endophytic strains BP328EF (*Codinaeopsis* sp.) and BP52EF (*G. moniliformis*) correlated strongly with stress indicators, specifically those related to primary photochemistry ABS/RC and DI_0_/RC), lipid peroxidation (MDA), leaf temperature, and petiole/leaf necrosis. As expected, the positive control plants (unprotected by endophytic fungi) were associated with high percentages of white mold symptoms, such as leaf wilting and flower bud necrosis, and exhibited elevated activities of oxidative metabolism enzymes (SOD, CAT, APX, and POD), signaling a state of physiological stress.

## 3. Discussion

### 3.1. Inoculation with Endophytic Fungi Mitigated the Deleterious Effects of the Phytopathogen S. sclerotiorum in Cotton Plants, Promoting Growth and Enhancing Physiological Performance

Our findings demonstrate that symbiosis with endophytic fungi can offset the damage caused by pathogen attack, thereby protecting cotton plants. Backman and Sikora [37] and Fontana et al. [38] proposed that the metabolic cost of biomass production for the endophyte is compensated by adaptive advantages, including a significant reduction in disease symptoms. In this study, strains BP16EF (*P. purpurogenum*), BP335EF (*G. moniliformis*), and BP33EF (*H. insecticola*) exhibited growth-promoting effects, as treated plants maintained or exceeded the stem height and diameter of the healthy control, even under pathogen pressure. Plant growth-promoting fungi frequently synthesize phytohormones, such as auxins, which stimulate vegetative development [39]. Furthermore, these fungi enhance nutrient uptake, providing plants with a competitive growth advantage [40,41].

Recent studies indicate that cotton plants may occlude xylem vessels as a structural defense mechanism known as tylosis [42]. Under *S. sclerotiorum* pressure, the maintenance or increase in stem diameter suggests the absence of atrophy or vessel loss; such structural integrity serves as evidence that the plant effectively bypassed or overcame the stress imposed by the phytopathogen [43,44]. Conversely, the unhindered progression of *S. sclerotiorum* hyphae through the stem typically leads to severe vascular obstruction and subsequent wilting [45]. Our results support the use of endophytic inoculation as a biotechnological tool to prevent vascular damage caused by this pathogen in cotton.

In this context, endophytic fungi proved essential for maintaining structural integrity, acting as a primary line of defense. This protection arises because these fungi occupy ecological niches similar to those of phytopathogens, allowing them to dominate the environment through niche competition, antagonism, mycoparasitism, or the induction of systemic resistance [46,47]. By enhancing host tolerance, endophytes promote growth and stress resilience through the production of bioactive compounds, including enzymes, alkaloids, and hormones, that are pivotal to the plant’s defense signaling [48,49].

### 3.2. Inoculation with Endophytic Fungi Mitigated Primary Photochemistry Stress by Minimizing Non-Photochemical Quenching and Maintaining Steady-State Carbon Fixation

Biotic stress in cotton plants lacking endophytic inoculation resulted in photochemical impairment, reduced photosynthetic rates, and low carboxylation efficiency. These findings support the premise that endophytic inoculation enhances stomatal conductance and carbon assimilation [50,51]. Rozpądek et al. [52] suggested that endophytic fungi improve water-use efficiency, allowing plants to utilize incident light for photochemistry more effectively through the up-regulation of genes related to photosystems I and II (PSI and PSII), as well as proteins involved in light harvesting and carbon fixation.

Alternatively, research indicates that plants release mucilage at the root tip that undergoes structural changes, shrinking and expanding, as the soil dries and re-wets [53]. This mucilage is partially degraded by microorganisms, some of which produce extracellular polymeric substances (EPSs) with properties similar to mucilage [54]. The resulting mucigel improves soil structure around the roots, increasing water retention and fostering a favorable environment for root development [55]. Consequently, this enhances water and nutrient uptake, which is reflected in the transpiration rates and stomatal conductance observed in inoculated plants [56,57]. Thus, the increased water absorption promoted by endophytes compensates for the maintenance of open stomata, leading to a greater net gain in photosynthesis [58]. Additionally, symbiotic fungal hyphae explore soil regions beyond the reach of roots, further increasing resource acquisition [59,60,61].

*S. sclerotiorum* colonizes the xylem of cotton, producing cottony mycelium and water-soaked lesions on petioles and leaves that progress to necrosis, wilting, and bud rot [45]. Extracellular enzymes, such as polygalacturonases, degrade pectin, a key cell wall component, during early infection [62]. Consequently, damaged leaves exhibit a reduced light-trapping capacity at the PSII reaction centers, increasing non-photochemical quenching (NPQ). Yang et al. [63] demonstrated that *S. sclerotiorum*-induced damage to the photosynthetic apparatus is driven by the synergistic effect of oxalic acid (H_2_C_2_O_4_) and oxalate ions (C_2_O_4_^2−^). Oxalate inhibits PSII activity, RuBP regeneration, and carboxylation efficiency, leading to the overproduction of reactive oxygen species (ROS). By inhibiting D1 protein synthesis, ROS further accelerate PSII photoinhibition. Overall, endophytic inoculation mitigated this photochemical stress, resulting in superior performance indices (PI_ABS_) in challenged plants. Furthermore, the increased electron transport flux (ET_0_/RC) indicates a robust capacity for light energy utilization, reflecting more efficient photosynthesis [64]. The lower energy dissipation in endophyte-treated plants suggests a more effective use of light energy, thereby minimizing energy losses [65,66].

We also observed that endophytic treatment helped maintain lower leaf temperatures; specifically, plants inoculated with BP10EF (*G. moniliformis*), BP16EF (*P. purpurogenum*), and BP33EF (*H. insecticola*) exhibited values consistent with low energy dissipation as heat (non-photochemical quenching, DI_0_/RC). In contrast, the elevated leaf temperatures in the positive control and in plants treated with BP335EF (*G. moniliformis*), BP52EF (*G. moniliformis*), and BP340EF (*Aspergillus* sp.) suggest vascular obstruction. Such obstruction impairs the upward water flow from roots to leaves [67], thereby driving the temperature increase. Furthermore, the restriction of water and nutrient transport induces oxidative stress, which alters water-use efficiency and raises local tissue temperatures due to energy imbalance [68,69]. This is corroborated by the low transpiration rates (*E*) observed in positive control plants, indicating a strict activation of stomatal closure to minimize water loss, a mechanism that inherently leads to increased leaf temperature [70,71].

### 3.3. Inoculation with Endophytic Fungi Modulated the Antioxidant Response, Ensuring Greater Cellular Homeostasis in Cotton Plants Stressed by S. sclerotiorum

The elevated activity of antioxidant enzymes (SOD, APX, POD, and CAT) in *S. sclerotiorum*-stressed plants lacking endophytic protection reflects a survival strategy against pathogen-induced damage [72]. Malenčić et al. [73] demonstrated that *S. sclerotiorum* infection induces an oxidative burst, triggering various antioxidant systems. The initial pathogenic stage involves oxalic acid production and the expression of cell wall-degrading enzymes, such as polygalacturonase isoforms (SSPG1) and proteases (ASPS), at the lesion’s expanding edge [74]. These activities release oligogalacturonides and peptides that induce a secondary wave of degradative enzymes, collectively resulting in the total dissolution of plant tissues.

Oxalic acid, alongside other metabolites, suppresses host defenses during the pathogenic phase, while specific components trigger host cell death, leading to necrosis [75]. Estoppey et al. [76] proposed that oxalotrophy, the microbial consumption of oxalic acid, is a viable biocontrol strategy against *S. sclerotiorum*. In this context, by reducing the pathogen’s colonization capacity, endophytes appear to mitigate ROS formation, allowing the plant to bypass the metabolic cost of activating an emergency antioxidant response [77,78,79]. During biotic stress, functional proteins are often overexpressed as part of enzymatic and non-enzymatic defense strategies [80]. This aligns with the high protein levels observed in the leaves of positive control plants, consistent with the increased synthesis of SOD, APX, and CAT.

The primary impact of ROS accumulation is the oxidation of signaling targets, including kinases, transcription factors (TFs), and stress-responsive proteins [81]. Consequently, plants affected by *S. sclerotiorum* require robust antioxidant synthesis to neutralize ROS and prevent uncontrolled hypersensitive responses. Endophytic microorganisms bolster the host’s defense state by modulating cellular redox homeostasis and reducing ROS accumulation [82,83]. Necrotrophic pathogens, such as *S. sclerotiorum*, rely on ROS-dependent mechanisms; for instance, *Botrytis*-induced necrosis and fungal development can be arrested by ROS inhibition [84]. Similarly, antioxidant pretreatment in barley has been shown to inhibit the development of the necrotrophic fungi *Pyrenophora teres* and *Rhynchosporium secalis* [85].

Overall, endophytic strains BP33EF (*H. insecticola*) and BP52EF (*G. moniliformis*) resulted in lower lipid peroxidation levels, revealing effective membrane protection strategies and highlighting the pivotal role of these fungi in the redox homeostasis of cotton plants challenged by white mold.

### 3.4. Inoculation of Cotton Plants with Endophytic Strains Mitigated S. sclerotiorum-Induced Biotic Stress, Preserving Anatomical Integrity and Minimizing the Severity of White Mold Symptoms

Inoculation preserved leaf tissues (epidermis and parenchyma), which were severely degraded in the positive control. This preservation stems from the biocontrol activity of the endophytes, leading to a drastic reduction in leaf wilting and flower bud necrosis. In practice, a thicker epidermis serves as an enhanced physical barrier, hindering penetration and limiting hyphal progression through leaf tissues [86]. Furthermore, epidermal thickening can mitigate water loss [87], a crucial strategy for preventing wilting in plants with vascular systems or organs colonized by pathogens. Notably, while epidermal thickening is a vital defense, it may not always suffice against necrotrophs that secrete cell wall-degrading enzymes and phytotoxins designed to breach such barriers.

Alternatively, a more developed palisade parenchyma may enhance light interception and photosynthetic efficiency [88,89], explaining the higher net photosynthetic rates (*A*) observed in inoculated plants. Corroborating these findings, inoculation with strains BP10EF (*G. moniliformis*), BP16EF (*P. purpurogenum*), BP33EF (*H. insecticola*), and BP340EF (*Aspergillus* sp.) resulted in thicker spongy parenchyma. This suggests that these strains assist the plant in managing water and biotic stress, given the parenchyma’s role in optimizing gas exchange and maintaining tissue turgor [90].

PCA confirmed a strong correlation between preserved leaf anatomy and low disease incidence, establishing strains BP10EF (*G. moniliformis*), BP16EF (*P. purporogenum*), and BP33EF (*H. insecticola*) as elite candidates for white mold management. This pioneering study evaluates these specific endophytic strains in the biocontrol of *S. sclerotiorum*; however, *G. moniliformis* has been previously identified as a producer of antimicrobial metabolites [91,92]. Furthermore, Silva et al. [25] demonstrated that *G. moniliformis* strains can express multiple plant growth-promoting traits, including phosphate solubilization and indoleacetic acid (IAA) synthesis.

Regarding *P. purpurogenum*, endophytic strains are known for their antimicrobial properties [93], highlighting the potential for prospecting metabolites such as polyketides [94,95], polyoxygenated bergamotanes, and monoterpenoids [96,97,98,99]. Li et al. [99] showed that antifungal extracts from *P. purpurogenum* can inhibit the necrotroph *Botrytis cinerea*, while Yuan et al. [100] reported that *P. simplicissimum* CEF-818 reduced the incidence of Verticillium wilt in cotton by 67% under greenhouse conditions. These findings underscore that *Penicillium* species produce bioactive compounds that serve as promising templates for new antifungals, reinforcing the genus’s potential for phytopathogen biocontrol [101].

Additionally, Silva et al. [26] recently reported the growth-promoting potential of the BP33EF de (*H. insecticola*) strain in cotton. Strains of this species produce high in vitro titers of metabolites such as avelanins A and B and tricinonoic acid [102]. Tricinonoic acid has demonstrated antibacterial activity [103] and was recently identified as a major non-volatile sesquiterpene in the metabolic profile of the biocontrol agent *Trichoderma longibrachiatum* [104]. In this study, we verified that the success of strains BP10EF (*G. moniliformis*), BP16EF (*P. purporogenum*), and BP33EF (*H. insecticola*) was not an isolated phenomenon, but rather an integrated physiological, biochemical, and anatomical response that culminated in the effective biocontrol of white mold and enhanced cotton plant performance.

Therefore, future research should focus on validating these results under field conditions to assess the persistence of these symbioses across diverse environmental and edaphoclimatic pressures. Furthermore, the capacity of these endophytes to mitigate oxidative stress and preserve the morpho-anatomical integrity of cotton paves the way for transcriptomic and metabolomic investigations. Such studies are essential to identifying the hormonal signaling pathways and specific secondary metabolites responsible for the observed physiological ‘shielding’. These advances will be fundamental to the development of multifunctional bio-inputs that integrate sustainable white mold management with the optimization of cotton crop productivity.

## 4. Materials and Methods

### 4.1. Preparation of Fungal Strains

Biotic stress mitigation assays were conducted using root endophytes isolated from the Arecaceae *B. purpurascens* [25], along with a phytopathogenic strain of *Sclerotinia sclerotiorum* from the microbial culture collection of the Agricultural Microbiology Laboratory at IF Goiano, Rio Verde campus, GO, Brazil. The fungal strains were plated on Potato Dextrose Agar (PDA) medium (infusion of 200 g potato, 20 g dextrose, and 15 g agar) and incubated for 7 days at 30 °C to obtain fresh cultures. The following endophytic strains were tested: BP10EF, BP335EF, and BP52EF (*Gibberella moniliformis*), BP16EF (*Penicillium purpurogenum*), BP328EF (*Codinaeopsis* sp.), BP33EF (*Hamigera insecticola*), and BP340EF (*Aspergillus* sp.). These strains were identified based on the sequencing of the ITS region [25] and selected based on their previously demonstrated potential to reduce the severity of ramulose in cotton plants [26]; the cultures belong to the strain bank of the Agricultural Microbiology Laboratory at IF Goiano, Rio Verde campus.

### 4.2. Plant Growing Conditions and Microbial Inoculation

The experiment was conducted under greenhouse conditions using cotton seeds of the cultivar FM912GLTP (FiberMax^®^ Cuiabá, Mato Grosso, MT, Brazil), recommended for the southwestern region of Goiás. Prior to sowing, the seeds were surface-disinfected following the procedure described by Reis et al. [105] to eliminate epiphytic microorganisms. After a 30 min resting period, the seeds were sown in soil previously autoclaved at 121 °C for 30 min and then stored in sealed, impermeable bags until planting. Seeds were sown at a depth of 2 cm in pots containing 3 kg of soil, with nine seeds per pot. Soil samples were collected in triplicate and subjected to chemical and physical characterization. The soil was classified as a dystrophic Red Latosol, with a particle size distribution of 44.0% sand, 12.0% silt, and 22.0% clay. The chemical properties of the 0–20 cm layer were as follows: pH (CaCl_2_) = 5.1; H + Al = 42 mmolc dm^−3^; P = 4 mg dm^−3^; K = 2.7 mmolc dm^−3^; S = 5.0 mg dm^−3^; Ca = 23 mmolc dm^−3^; Mg = 11 mmolc dm^−3^; cation exchange capacity = 78.7 mmolc dm^−3^; Cu = 2.8 mg dm^−3^; Fe = 28 mg dm^−3^; Mn = 11.9 mg dm^−3^; Zn = 0.5 mg dm^−3^; and B = 0.13 mg dm^−3^. Based on these results, soil chemical and nutritional attributes were corrected with macro- and micronutrients, following the recommendations of Sousa and Lobato [106]; additionally, soil acidity was corrected using calcitic limestone (PRNT = 90%) at a rate of 2.5 t ha^−1^ to increase base saturation to 70%.

Endophytic fungal inocula were prepared on PDA medium, and at sowing, 5 mm mycelial disks were placed in direct contact with the seeds to allow simultaneous development of fungal hyphae and seedling radicles. Greenhouse temperature and relative humidity were monitored throughout the experiment, with mean values of 30.23 °C and 28.49%, respectively. Pots were inspected daily and irrigated as required.

Fifteen days after emergence, seedlings were thinned to two plants per pot. One removed plant per pot was used to confirm endophytic colonization through re-isolation of fungi from root fragments. Two months after emergence, at the R3 growth stage, cotton plants were inoculated with the phytopathogen. The *S. sclerotiorum* strain was cultured in Czapek–Dox broth (Kasvi^®^, São José dos Pinhais, Paraná, PR, Brazil) under agitation (90 rpm) for 6 days at 30 °C. The culture was filtered to remove mycelium, and the conidial concentration of the resulting suspension was adjusted to 1 × 10^7^ conidia mL^−1^ using a hemocytometer. Inoculation was performed by applying 100 µL of the spore suspension into the soil at a depth of 2 cm and 2 cm from the root collar. This inoculation method was chosen to simulate natural infection through myceliotrophic colonization, which occurs from sclerotia present in the soil. Plants were maintained for 90 days until reaching the R6 phenological stage (peak flowering), during which growth, physiological, stress-related metabolic, anatomical, and disease incidence parameters were evaluated.

### 4.3. Growth and Physiological Assessments

Plant height (cm) and stem diameter (cm) were recorded. All physiological, antioxidant metabolism, and anatomical analyses were performed using the third fully expanded leaf from the plant apex. Chlorophyll *a*, *b*, and total chlorophyll contents were estimated using a portable chlorophyll meter (ClorofiLOG^®^ 2060, Falker^®^, Porto Alegre, RS, Brazil), and results were expressed as the Falker Chlorophyll Index (FCI).

Chlorophyll *a* fluorescence induction (OJIP transient) was measured using a portable FluorPen FP 100 fluorometer (Photon Systems Instruments, Drasov, Czech Republic). Prior to measurement, the third leaf of each experimental unit was dark-adapted for 30 min to ensure complete oxidation of the photosynthetic electron transport chain. Leaves were then exposed to a saturating pulse of blue light (3000 µmol m^−2^ s^−1^), allowing for determination of minimum fluorescence (F_0_) at 50 µs, corresponding to step O with all PSII (photosystem II) reaction centers open, followed by steps J (2 ms) and I (30 ms), and maximum fluorescence (F_m_), corresponding to step P, when all PSII reaction centers were closed. These parameters were used to calculate PSII bioenergetic indices according to Strasser et al. [107], including the absorption flux per reaction center (ABS/RC), trapped energy flux per reaction center (TR_0_/RC), electron transport flux per reaction center (ET_0_/RC), maximum quantum yield of primary photochemistry (PHI_P0_), photosynthetic performance index (PI_ABS_), and energy dissipation flux per reaction center (DI_0_/RC).

Leaf temperature was measured by infrared thermography using a FLIR E60 camera (thermal sensitivity < 0.05 °C; accuracy ± 2 °C). Images were captured at a fixed distance of 30 cm from the adaxial leaf surface, with leaves positioned horizontally and the measurement point centered on the leaf blade. Thermal images and temperature data were recorded for subsequent analysis.

Gas exchange measurements were performed using an infrared gas analyzer (IRGA) equipped with a fluorometer (LI-6400XT, LI-COR Inc., Lincoln, NE, USA). The following parameters were determined: net CO_2_ assimilation rate (*A*, µmol CO_2_ m^−2^ s^−1^), transpiration rate (*E*, mol H_2_O m^−2^ s^−1^), internal CO_2_ concentration (*Ci*, µmol m^−2^ s^−1^), and stomatal conductance to water vapor (Gsw, mol H_2_O m^−2^ s^−1^). Water use efficiency was calculated as the *A/E* ratio, and carboxylation efficiency as *A/Ci*. Measurements were conducted between 07:00 and 11:00 h under constant photosynthetically active radiation (PAR; 1000 µmol photons m^−2^ s^−1^), ambient CO_2_ concentration, and prevailing temperature and humidity conditions.

### 4.4. Activity of Antioxidant Metabolism Enzymes, Proline and Malondialdehyde (MDA)

The activities of antioxidant enzymes, proline accumulation, and lipid peroxidation were quantified. Leaf samples were collected, immediately frozen in liquid nitrogen, and stored at −80 °C until analysis. Enzyme extraction was performed by macerating 200 mg of fresh leaf tissue in liquid nitrogen with 50% (*w/w*) polyvinylpolypyrrolidone (PVPP), following the protocol described by Biemelt et al. [108]. The extraction buffer consisted of 100 mM potassium phosphate (pH 7.8), 0.1 mM EDTA, and 10 mM ascorbic acid. The homogenate was centrifuged at 13,000× *g* for 10 min at 4 °C, and the supernatant was used to determine the activities of catalase (CAT), ascorbate peroxidase (APX), guaiacol peroxidase (POD), and superoxide dismutase (SOD).

CAT activity was determined according to Havir and McHale [109] by monitoring the decrease in absorbance at 240 nm due to H_2_O_2_ consumption. The reaction mixture contained 100 mM potassium phosphate buffer (pH 7.0) and 12.5 mM H_2_O_2_, and absorbance was recorded every 15 s for 3 min. An extinction coefficient of 36 mM^−1^ cm^−1^ was used, and CAT activity was expressed as µmol H_2_O_2_ min^−1^ mg^−1^ protein.

APX activity was assayed following Nakano and Asada [110] by measuring the oxidation of ascorbate at 290 nm every 15 s for 3 min. The reaction medium consisted of 100 mM potassium phosphate buffer (pH 7.0), 0.5 mM ascorbic acid, and 0.1 mM H_2_O_2_. An extinction coefficient of 2.8 mM^−1^ cm^−1^ was used, and APX activity was expressed as µmol ascorbate (AsA) min^−1^ mg^−1^ protein. POD activity was determined according to Fang and Kao [111] by monitoring tetraguaiacol formation at 470 nm. The reaction mixture contained 50 mM sodium phosphate buffer (pH 6.0) and 0.13% guaiacol, and the reaction was initiated by adding 0.15% H_2_O_2_ (3 min). Absorbance was recorded using an extinction coefficient of 26.6 mM^−1^ cm^−1^. POD activity was expressed as µmol H_2_O_2_ min^−1^ mg^−1^ protein.

SOD activity was measured following Giannopolitis and Ries [112], based on the enzyme’s ability to inhibit the photoreduction of nitroblue tetrazolium (NBT). The reaction mixture contained 50 mM potassium phosphate buffer (pH 7.8), 14 mM methionine, 0.1 µM EDTA, 75 µM NBT, and 2 µM riboflavin. Samples were illuminated under a 20 W fluorescent lamp for 7 min, and absorbance was read at 560 nm. One unit of SOD activity was defined as the amount of enzyme required to inhibit NBT photoreduction by 50%, and results were expressed as U mg^−1^ protein.

Lipid peroxidation was estimated by quantifying malondialdehyde (MDA) following the method of Buege and Aust [113]. Leaf tissue (200 mg) was macerated in liquid nitrogen with PVPP, homogenized in 0.1% (*w*/*v*) trichloroacetic acid (TCA), and centrifuged at 10,000× *g* for 15 min at 4 °C.

Total soluble protein content was quantified using the Bradford method [114], with absorbance measured at 595 nm, and used to normalize enzymatic activities.

Proline concentration was determined according to Bates et al. [115]. Dry leaf tissue (100 mg) was extracted with 3% sulfosalicylic acid, homogenized at room temperature for 60 min, and filtered. Aliquots of the filtrate were reacted with acid ninhydrin and glacial acetic acid (2 mL each) and incubated at 100 °C for 1 h. Absorbance was measured at 520 nm, and proline concentration was calculated using a standard curve.

### 4.5. Characterization of Epidermal and Mesophyll Development

Leaf samples were taken by making 3 cm^2^ cuts from the central region of the leaf. Subsequently, these were fixed in Karnovsky’s solution [116] for 24 h. After this period, the plant material was pre-washed in phosphate buffer (0.1 M, pH 7.2) and dehydrated in an increasing ethyl series (30% to 100%), pre-infiltrated and infiltrated in historesin (Leica, Wetzlar, Hessen, Germany), according to the manufacturer’s recommendations. The samples were sectioned transversely at 5 μm thickness using a rotary microtome (Model 1508R, Logen Scientific, Jinhua, Zhejiang, China). For structural analysis, the sections were stained with toluidine blue—polychromatic staining (0.05% 0.1 M phosphate buffer, pH 6.8) [117]. Morphoanatomical observations of the epidermis on the adaxial and abaxial surfaces, palisade parenchyma, and spongy parenchyma were performed. Measurements of the thicknesses of the different anatomical layers were performed using ImageJ software, version 1.54r [118].

### 4.6. Incidence of Plants Affected by the Phytopathogen

The incidence of white mold was assessed by the percentage of cotton plants exhibiting typical symptoms [4]. The symptoms evaluated were: leaf wilting: loss of turgor during the day, but without recovery at night; petiole and leaf rot: presence of brown or light brown necroses that may or may not have evolved into wet rot of grayish color, covered by white and cottony mycelium of the fungus; flower bud rot: soft flower buds, exhibiting wet rot and emission of white and cottony mycelium.

### 4.7. Experimental Design and Statistical Analyses

The experiment was conducted in a randomized block design with nine treatments: seven endophytic fungal strains, a negative control (healthy, non-inoculated plants without endophytic fungi), and a positive control (plants inoculated with *S. sclerotiorum* but not treated with endophytic fungi). Each treatment was evaluated with five replicates (five pots per treatment), with two plants per pot. Data were subjected to analysis of variance (ANOVA), considering each pot as an independent unit, and treatment effects were assessed using the *F*-test at the 5% probability level. When significant effects were detected, mean comparisons were performed using the Scott–Knott test (*p* < 0.05). In addition, the individual effects of each treatment were compared with the positive control using Dunnett’s test (*p* < 0.05). Subsequently, all variables were jointly evaluated in a correlation matrix and connected by means of principal component analysis (PCA). Considering that these variables had different units of measurement, PCA was recovered using standardized data to obtain a mean of 0 and a standard deviation of 1. The number of principal components was defined according to eigenvalues (>1.0) and variance explained (>80%). All statistical analyses were performed using the R software environment, 4.5.1 version [119].

## 5. Conclusions

We confirmed the hypothesis that endophytic fungal strains isolated from the roots of an endemic Cerrado plant can effectively control white mold symptoms in cotton. Overall, inoculation with strains BP10EF (*G. moniliformis*), BP16EF (*P. purpurogenum*), and BP33EF (*H. insecticola*) acted as a potent physiological modulator, mitigating the deleterious effects of *S. sclerotiorum* infection. These benefits were evidenced by the maintenance of vegetative vigor and the preservation of the photosynthetic apparatus. Notably, strain BP33EF (*H. insecticola*) ensured high photosynthetic performance indices (PI_ABS_) and carboxylation efficiency (*A/Ci*), even under pathogen pressure. Beyond promoting growth in height and diameter, these strains prevented the structural collapse of leaf tissues by preserving the thickness of the palisade and spongy parenchyma. This structural integrity resulted in optimized leaf temperature regulation and near-total suppression of leaf wilting and reproductive organ necrosis. The symbiosis established by the fungal cluster BP10EF (*G. moniliformis*), BP16EF (*P. purpurogenum*), and BP33EF (*H. insecticola*) promoted superior cell membrane stability, reflected in low MDA levels and reduced antioxidant enzyme activity (SOD, APX, and CAT). This suggests that these endophytes prevent severe oxidative stress, ensuring homeostatic maintenance before the oxidative burst compromises plant health.

## Data Availability

All the data relevant to this manuscript are available on request from the corresponding author.
